# Genetic Status of the Northernmost Population of the Endangered and Elusive Huemul Deer, *Hippocamelus bisulcus*

**DOI:** 10.3390/ani16111727

**Published:** 2026-06-04

**Authors:** Juan C. Marín, Carlos Venegas, Gonzalo Flores Morales, Andrés Peña Monroy, Juan Pablo Vásquez, Rodrigo Andrés López Rübke, Ana Carolina Hinojosa Sáez, Alexandra Chávez, Warren E. Johnson, Pablo Orozco-terWengel

**Affiliations:** 1 Laboratorio de Genómica y Biodiversidad, Facultad de Ciencias, Universidad del Bío-Bío, Chillán 3810189, Chile; caralvenca@gmail.com (C.V.); gonzalof759@gmail.com (G.F.M.); andres.pena0526@gmail.com (A.P.M.); jnpablov@gmail.com (J.P.V.); alexandra.chavez@uni-mainz.de (A.C.); 2 Aumen, El Eco de los Montes (NGO), 18 de Septiembre 629, Coyhaique 5950000, Chile; lopezrubke@gmail.com; 3 Departamento de Áreas Silvestres Protegidas, Corporación Nacional Forestal (CONAF), Chillán 3800773, Chile; ana.hinojosa@conaf.cl; 4 Department of Biology, Loyola University Maryland, Baltimore, MD 21210, USA; johnsonw11661@gmail.com; 5 School of Biosciences, Cardiff University, Cardiff CF10 3AX, UK

**Keywords:** conservation genetics, small population, microsatellites, South America

## Abstract

Small, isolated, and fragmented populations often exhibit low levels of genetic diversity as a result of genetic drift, limited gene flow, and inbreeding. Huemul (*Hippocamelus bisulcus*) is a medium-sized South American deer categorized as endangered by the International Union for Conservation of Nature (IUCN). Huemul in its northernmost range was previously distributed in Central Chile between 36° S and 37° S, but its current distribution and conservation status in the region are poorly documented. We used non-invasive genetic approaches to assess the genetic diversity, population connectivity, and demographic history of the huemul’s northernmost population using nuclear microsatellite markers from fecal samples of wild individuals. Observed nuclear DNA genetic variation (Ho = 0.2958 ± 0.0318) was moderate, and allelic richness was low (Ar = 3.43–4.01), consistent with the theoretical expectation that isolated populations may retain heterozygosity while losing allelic richness more rapidly. However, the estimated nuclear DNA effective population size was low (Ne = 47; 95% CI: 19.2–∞). Demographic simulations project continued loss of genetic diversity under all scenarios modeled. Our results provide a foundation for further study of this population and provide the genetic data necessary to design detailed management plans to ensure the persistence of healthy populations of this rare and elusive deer.

## Simple Summary

The huemul is an endangered deer that lives only in the Southern Andes of Chile and Argentina. Once widespread, fewer than 2000 individuals survive in scattered, isolated groups. The northernmost population—~50 deer in Central Chile’s Nevados de Chillán mountains—is separated from its nearest neighbors by 400 km of unsuitable habitat, leaving it especially vulnerable to extinction. We collected huemul fresh feces across the northernmost population’s entire range and used nuclear DNA to ask (i) how much genetic diversity remains in this population, (ii) whether the deer are mating with relatives, and (iii) what the effective population size of this population is. We identified 36 unique animals and found that, although they retain a moderate level of genetic variation, mating among relatives is unusually high and the effective population size is below 50. Computer simulations show that, without action, this population will continue to lose genetic diversity and may disappear within decades. Our results provide the first genetic baseline for monitoring this population and support urgent conservation measures, including protecting habitat corridors between Chillán and Laguna del Laja, safeguarding private lands threatened by mining and infrastructure projects, and coordinating efforts with Argentina to preserve this iconic Andean deer.

## 1. Introduction

Small and geographically isolated populations of endangered ungulates are particularly vulnerable to the effects of genetic drift, inbreeding, demographic and environmental stochasticity, all of which reduce their adaptive potential and increase extinction risk [1,2]. In South America, habitat degradation and fragmentation caused by human activities have intensified these threats for several cervid species, particularly those with narrow ecological requirements or naturally fragmented ranges [3,4]. These processes are especially concerning in species such as the huemul (*Hippocamelus bisulcus*), a forest and mountain deer endemic to the Southern Andes, where its populations persist in small numbers and under increasing isolation [5]. For this species, previous mitochondrial DNA research has highlighted the unique evolutionary trajectory and severe genetic isolation of its northernmost population in Central Chile [6]. In such contexts, assessing genetic diversity and connectivity becomes a fundamental conservation tool to determine whether isolated populations retain the genetic viability necessary for long-term persistence [7]. In addition, non-invasive genetic sampling offers a minimally disruptive method for investigating these parameters in elusive or threatened species [8,9].

The huemul is South America’s southernmost distributed endemic deer species, with isolated populations distributed across Chile and Argentina between 36° S and 54° S. Huemul occupy a variety of mountainous terrains, including open forested habitats, areas of low precipitation in Central Chile, sub-Antarctic rainforests, and periglacial coastal habitats in the Patagonian fjords [10]. Extant populations are largely fragmented into small, isolated subpopulations [6], and as such, the IUCN has classified huemul as endangered [11]. Population fragmentation is likely the response to local habitat loss and fragmentation [12,13], poaching and attacks by domestic dogs [14,15,16], and population size reductions possibly exacerbated by the introduction of exotic livestock carrying diseases over the last 200 years [17,18]. Currently, the huemul population is estimated to be between 1000 and 2000 animals, with most individuals inhabiting the more southern parts of Chile and Argentina [6,10,13] and a small population of approximately 50 individuals occurring around Nevados del Chillán [19].

Nevados de Chillán is characterized by arid terrain with mixtures of steep slopes (30–90°) and intermittent flat plateaus < 20° [20] where snow accumulation tends to be low, and with altitude ranging between 1600 and 2100 masl [21]. This area is characterized by moist meadows with suitable plants to browse, including annual herbs, patches of ñirre (*Nothofagus antarctica*) and lenga (*Nothofagus pumilio*). Huemul often shelter in the area in patches of paramela (*Adesmia boronioides*) and prickly heath (*Gaultheria mucronate*) that border rivers, streams and other water bodies [20]. Relatively recent studies recognized 15 sites in Nevados de Chillán with resident populations of huemul, representing ~35% of the species’ original distribution in this area [19,22]. The huemul population of Nevados de Chillán is of evolutionary and conservation relevance as it is likely a refugial population with a unique biogeographic history [6]. The area between Nevados de Chillán and Laja Lagoon in Central Chile is an important and unique biodiversity area that harbors high numbers of endemic species known to have experienced important climatic and ecological changes during and after the Last Glacial Maximum [23]. In the last decade, the Chilean Forest and Park Service (CONAF) developed the Huemul Recovery, Conservation and Management Plan for this area [24], registering animals using camera traps in the two state-protected reserves in the region and in several privately managed forest areas (e.g., Huemules del Niblinto National Reserve, Ñuble National Reserve and Laguna del Laja National Park–Hinojosa-Sáez pers. comm.).

A previous genetic study of the northernmost huemul population of Nevados de Chillán, conducted using mitochondrial DNA (mtDNA) markers [6], identified this population as a genetically distinct Central Chile cluster, characterized by the lowest haplotypic diversity among all sampled clusters, signatures of recent demographic expansion (~6000 years BP), and extreme geographical isolation (~400 km from the nearest population at Nahuel Huapi National Park [6]). Furthermore, a significant pattern of isolation-by-distance across the species’ range (r = 0.377, *p* = 0.001) and the highest pairwise differentiation values involving this cluster (ΦST = 0.829) suggest that the Nevados de Chillán population may have originated from a small number of founders derived from Northern Patagonia, with subsequent restricted gene flow reinforcing its genetic distinctiveness. However, mtDNA provides a single maternally inherited locus with limited resolution for assessing contemporary connectivity, inbreeding, and fine-scale population structure. Here we use nuclear microsatellite markers to (i) determine whether genetic variation has been eroded as a consequence of prolonged isolation, (ii) identify signals of recent or historical connectivity among individuals and sampling sites, and (iii) explore whether demographic fluctuations have left detectable signatures in the genetic makeup of the population. These insights are essential to inform management strategies and evaluate the long-term viability of this peripheral huemul population in the context of broader species conservation planning.

## 2. Materials and Methods

### 2.1. Sample Collection

**Figure 1:**
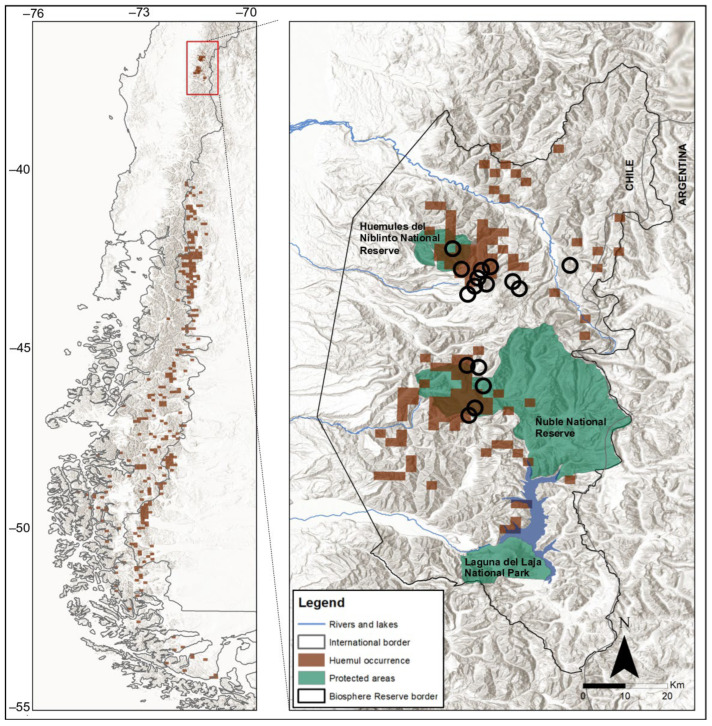
Map depicting the distribution range of huemul with an expansion of the study area in the Nevados de Chillán. Brown squares indicate the presence of huemul, and the circles show the sampling localities of this study.

During the summers and autumns of 2008 to 2010, 49 fresh huemul feces were collected in Huemules del Niblinto National Reserve, Ñuble National Reserve and in their surroundings (Table 1, Figure 1). A sample was considered to be fresh (less than one day-old) if it was glossy, had a dark color and was covered with mucus. The GPS coordinates of samples were recorded, and samples were preserved in 30 mL of absolute ethanol until arrival at the Genomics and Biodiversity Laboratory of the Universidad del Bío-Bío (Chillán, Chile), where they were stored at −80 °C. A total of 829.3 km^2^ was surveyed, representing ~27% of the distribution of huemul in Central Chile. Samples were collected from 17 localities with an average of two samples per sector; some of which were discarded from this analysis due to the low quality of the extracted DNA. Sampling followed the guidelines of the American Society of Mammologists [25]. Additionally, as home ranges of huemul average around 350 to 444 ha [5,26], we collected samples no less than 1 km apart from each other to minimize the risk of sampling the same individual twice. Total genomic DNA was extracted using the QIAGEN QIAamp^®^ DNA Stool Mini (Qiagen, Hilden, Germany) kit following the manufacturer’s instructions.

### 2.2. Microsatellite Analysis

Size variation in seven autosomal dinucleotide microsatellite loci specifically designed for huemul [27] was analyzed. PCR reactions consisted of 10 ng of total DNA, 2.5 µL of 10× buffer gold, 200 mM of each dNTP, 10 pm of each primer [with the forward primer fluorescently labeled], 2 mM MgCl_2_ and 1.5 U of AmpliTaq Gold polymerase (Applied Biosystems, Foster City, CA, USA) in a total volume of 25 µL. PCR reactions were performed with a starting temperature of 95 °C for 15 min, followed by 35 cycles of 94 °C for 30 s, 60 °C for 90 s and 72 °C for 60 s with a final extension at 60 °C for 30 min. All analyses included positive and negative controls to be able to avoid random shifts in fragment size between runs and to minimize the generation of false alleles. Fragments were analyzed on an ABI Prism^®^ 3100 Genetic Analyzer (Applied Biosystems) and sized using Genemarker v1.70 (SoftGenetics, State College, PA, USA) software. Amplification and genotyping of DNA from fecal samples were repeated two or more times to ensure repeatability of the genotypes. We evaluated duplicated samples by means-matching microsatellite genotypes using Microsatellite Toolkit [27] and eliminated samples from the study if they showed a complete allele match between at least six of seven microsatellite loci (85% overlap). The repeated samples (*n* = 3), as well as the samples that did not amplify correctly (*n* = 10), were eliminated from subsequent analyses. Additionally, unique individual genotypes were identified from the remaining samples using the allelematch package in R v4.6.0 [28], which clusters multilocus profiles while explicitly accommodating missing data and potential genotyping errors. To validate the discriminatory power of our marker panel within a naturally structured population, we calculated the cumulative probability of identity (P_ID_) and probability of identity among siblings (P_IDsib_) by sequentially adding loci from most to least informative [29,30]. We applied the conservative threshold of P_IDsib_ < 0.05 to ensure the reliable differentiation of closely related individuals. We also evaluated the existence of null alleles using the program Micro-Checker v. 2.2.3 [31]. Hardy–Weinberg equilibrium and linkage disequilibrium between loci were tested using GENEPOP v4.7.5 [32].

General microsatellite diversity statistics, including the number of observed alleles (N_A_), number of effective alleles (N_EA_), the N_EA_/N_A_ ratio, allelic richness (Ar) and Shannon’s Information Index (I), average number of alleles per locus (A), expected heterozygosity (He), and observed heterozygosity (Ho) were calculated using the poppr [33] and hierfstat [34] packages in R. The inbreeding coefficient (F_IS_) and its significance were also estimated using 15,000 bootstrap replicates within these packages.

Due to the relative isolation of the huemul population of Central Chile from the other Patagonian populations, we tested whether there was evidence of bottleneck events and/or a decrease in effective population sizes (Ne). We used the program Bottleneck v.1.2.0.2 [35] to test for the presence of a transitory excess of heterozygosity for the number of alleles observed, which is the hallmark of a bottleneck [36]. Bottleneck was run assuming the stepwise mutation model (SMM) and the multiple-step stepwise mutation model (TPM). Under the TPM, the proportion of single-step mutation events was set at 90% with a mutation size variance of 12%. Expected heterozygosity was compared with the expected heterozygosity for each locus, given the observed number of alleles, using a Wilcoxon signed-rank test [35].

Genetic structure was determined with STRUCTURE v2.3.3 [37], using 10 runs consisting of a burn-in of 200,000 steps of the Markov Chain Monte Carlo (MCMC) algorithm and a collection phase of 1,000,000 steps, with K (number of groups) values of 1 through 6. Default settings were used for the other run parameters, except when allele frequencies were determined to be correlated. The most likely K was determined using the DeltaK approach as implemented in StructureSelector [38]. To visualize and estimate spatial patterns of population connectivity, we used the Estimated Effective Migration Surface (EEMS) method [39]. We ran 10 independent chains, each with 10 million MCMC iterations, a burn-in of 100,000 iterations, 100 demes, and a thinning interval of 9999. Chain convergence was confirmed using rEEMSplots; no lack of convergence was detected. The population pairwise F_ST_ was calculated between clusters using the hierfstat package with 999 random permutations to estimate significance.

The effective population size (Ne) was calculated using NeEstimator v.2 [40], with an allele frequency cutoff of 0.01 and a confidence interval [CI] of 95% using the Jackknife method [41]. To assess the effect of genetic drift, simulations were conducted using BottleSim v2.6.1 [42] under different scenarios, with the following parameters: lifespan = 15 years, age at maturity = 3 years, completely overlapping generations, random mating, dioecious reproduction, and sex ratio of F:M: 1.5:1 and 1000 iterations. Genetic diversity simulations were carried out over 100 years, retaining 100, 90, 75, 50, and 25% of an expected census population size (Nc) of 50 deer, as the current Nevados de Chillán population.

## 3. Results

### 3.1. Microsatellite Diversity

**Figure 2:**
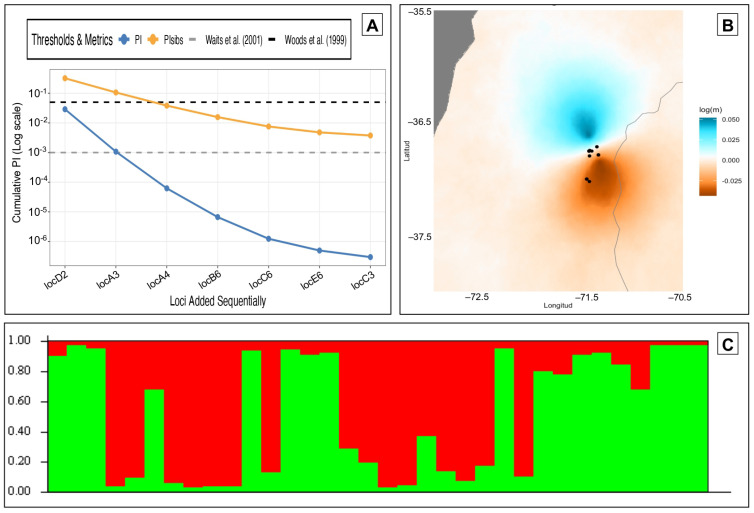
(**A**) Cumulative probability of identity for unrelated individuals (P_ID_ in blue) and for siblings (P_IDsib_ in orange). The P_ID_ threshold of <0.001 suggested by Waits et al. [29] (gray dashed line) and the P_IDsib_ threshold of <0.05 suggested by Woods et al. [30] (black dashed line) are included. Loci are added to the combinations in order from the most to the least informative. (**B**) An effective estimate of migration surface showing higher migration on the north of the Nevados de Chillan range (light blue) and less migration on the south of the range (brown). (**C**) Barplot from the analysis of population structure showing the two huemul groups identified with seven microsatellites.

Of the 49 huemul samples collected, DNA could be extracted and microsatellite genotyping performed for 36. Multilocus clustering determined that these samples correspond to 36 different individuals. The 7-locus panel provided robust discrimination capacity (cumulative P_ID_ = 2.97 × 10^−7^; cumulative P_IDsib_ = 0.00376; Figure 2A). This P_IDsib_ value is an order of magnitude below the established 0.05 threshold for non-invasive capture-mark-recapture studies [31], confirming sufficient statistical power to accurately distinguish individuals, even within a hypothetical population comprised entirely of full siblings. Six loci deviated from the Hardy–Weinberg equilibrium due to an excess of homozygotes (*p*-value: 0.001), and no linkage disequilibrium was found between loci. Analysis with Micro-Checker detected evidence of null alleles in five loci (locA3, locA4, locD2, locC3, and locB6), with estimated frequencies ranging from 0.19 to 0.42 (van Oosterhout method). The locus locD2 exhibited the highest null allele frequency (0.42). Only locE6 showed no evidence of null alleles. A total of 48 alleles were found across the seven microsatellites, with the number of alleles in a locus varying between 3 (E6) and 12 (D2) and with an average number of alleles per locus of 6.86 (N_A_) and a global allelic richness (Ar) of 6.50 per locus. General microsatellite diversity statistics revealed a mean number of effective alleles (N_EA_) of 4.21. Consequently, the overall N_EA_/N_A_ ratio was 0.59. Shannon’s Information Index (I) for the overall population was 3.58. The observed heterozygosity (H_O_) averaged 0.2958 ± 0.0318, and the expected heterozygosity (H_E_) averaged 0.6353 ± 0.0568. The average inbreeding coefficient (F_IS_) across loci was 0.5663 ± 0.01636 and significantly higher than zero (*p* < 0.001).

### 3.2. Population Structure, Connectivity, and Effective Population Size

The EEMS analysis (Figure 2B) revealed a pronounced north vs. south divide, with the northern portion of the range supporting higher animal movement, whereas the southern portion showed markedly reduced connectivity. The analysis of population structure identified K = 2 as the best model to group the samples (Figure 2C), with the two groups showing varying levels of admixture between their members. The pairwise divergence distance F_ST_ between the two clusters was 0.149 (*p* < 0.001, 95% CI = 0.041–0.279), indicating statistically significant and moderate genetic differentiation between the two groups.

**Figure 3:**
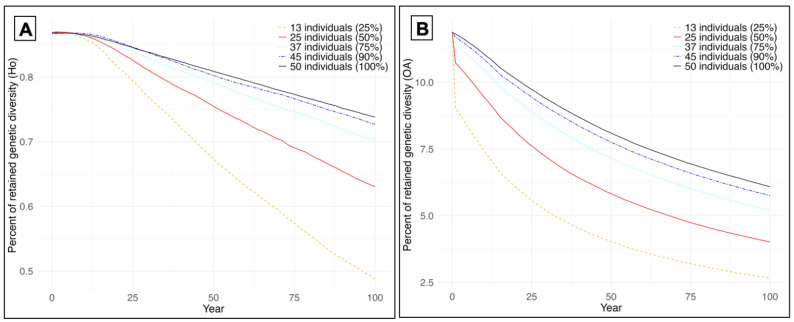
Projected retention of observed heterozygosity (**A**) and observed allele number (**B**) over 100 years in the huemul (*Hippocamelus bisulcus*) population, assuming a census size (Nc) of 50 individuals and a F:M sex ratio of 1:1. Projections were obtained using BOTTLESIM v2.6 under four scenarios retaining 100% (blue dash–dot line), 90% (cyan dotted line), 75% (red solid line), and 50% (yellow dashed line) of the current population size. Both genetic diversity measures show a progressive decline over time, with faster losses at lower retained population sizes.

The analyses with the software Bottleneck found no excess of expected heterozygosity given the number of alleles observed in the Nevados de Chillán huemul population, indicating that there is no evidence of a genetic bottleneck in the recent history of this population (Wilcoxon signed rank test TPM *p*-value: 0.28906 and SMM *p*-value: 0.46875, respectively). The Ne estimates were low, with a mean of 47 individuals and a 95% confidence interval ranging between 19.2 and infinity. The BottleSim simulations resulted in a decline in the observed heterozygosity (Figure 3A) and observed number of alleles (Figure 3B) under all simulated scenarios, indicating that a census population size of 50 is not sufficient to avoid the systematic loss of genetic diversity over time. When the census population falls below 50 reproducing adults, genetic variation is lost at a substantially faster rate.

Furthermore, the loss of the observed number of alleles (OA; Figure 3B) was more rapid than the loss of observed heterozygosity (H_O_), and a retention of 75% of the starting population is necessary to avoid a loss of more than 50% of the allelic diversity (a loss of 30% of H_O_) across 100 years.

## 4. Discussion

In this study, we analyzed for the first time huemul non-invasive samples collected in the Nevados de Chillán area, the northernmost distribution range of the species. From the 49 samples collected, we generated microsatellite genotypes for 36 samples. None of the samples corresponded to the same individual sampled multiple times. As with previous demographic models [5,6,19,43,44], our results reinforce the need for more detailed population modeling and conservation assessments of this species, especially because of the lack of connectivity between this northern population and other neighboring populations.

### 4.1. Genetic Diversity and Inbreeding

The Nevados de Chillán population has similar levels of diversity to those seen in other more numerous and better-connected huemul populations in Patagonia [6]. Despite having a high inbreeding coefficient, the Central Chilean huemul population had a high expected heterozygosity for such a small and isolated population. In fact, the Nevados de Chillán population shows heterozygosity levels (H_O_: 0.2906; H_E_: 0.6353) higher than those observed in the much larger Patagonian huemul populations [5]. However, the inbreeding coefficient (F_IS_) was much higher in the Nevados de Chillán population when compared to the Patagonian huemul populations (0.563 vs. 0.009, respectively). The high F_IS_ may be partly influenced by the presence of null alleles detected in five of seven loci, which can inflate homozygosity estimates. However, the concordance between multiple genetic indicators—including low effective population size, reduced connectivity inferred from EEMS analysis, and population structure suggesting limited gene flow—suggests that inbreeding is also a significant factor shaping the Nevados de Chillán huemul population genetic diversity. However, this observation may also be the outcome of the Wahlund effect, where our large F_IS_ estimate derives from the simultaneous analyses of individuals that descend from two separate ancestries, as inferred from the analysis of population structure. While high F_IS_ may be partly influenced by the presence of null alleles, it is highly likely that a combination of ecological and demographic factors is driving this pattern. The severe habitat fragmentation in central Chile, driven by land-use changes, limits dispersal and drastically reduces gene flow between the remaining huemul subpopulations [13,19]. Coupled with historical bottlenecks and a persistently small census population size, these conditions inevitably lead to mating among closely related individuals [5,45], thereby increasing inbreeding levels well beyond what is observed in the larger, more continuous Southern Patagonian huemul populations [5,6].

The moderate but highly significant genetic differentiation observed between the two sub-clusters (F_ST_ = 0.149, *p* < 0.001) corroborates the spatial discontinuity identified by the EEMS analysis. This distinct subdivision strongly supports the hypothesis of an underlying Wahlund effect. Furthermore, our extended diversity metrics provide deeper insights into the genetic architecture of these clusters. The northern cluster exhibited slightly higher allelic richness (Ar = 4.01) compared to the southern cluster (Ar = 3.43). More importantly, the ratio of effective to observed alleles (N_EA_/N_A_) was closer to one in the northern cluster (0.85 vs. 0.73), indicating a more even distribution of allele frequencies. This suggests that while the southern cluster is experiencing a more acute effect of genetic drift—likely driven by stronger isolation and smaller local population sizes—the northern group may still retain a more stable genetic composition. Together, these metrics emphasize that genetic erosion and fragmentation are not uniform across the Nevados de Chillán landscape, reinforcing the need for spatially explicit conservation interventions.

Nevertheless, relative to other South American ungulate populations—most notably the Bolivian guanaco, which has a comparably small and isolated population—the Nevados de Chillán huemul shows only moderate levels of inbreeding [5]. Moreover, similar results have been described in genetically healthy populations of other species, such as the forest musk deer *Moschus berezovskii* (F_IS_ = 0.317–0.357) [46] and in marsh deer *Blastoceros dichotomus* (F_IS_ = 0.163) [47,48]. To provide a broader context, the genetic diversity observed in the Nevados de Chillán huemul population can also be compared with managed populations of other cervids. For instance, farmed sika deer (*Cervus nippon*) exhibit specific patterns of genetic diversity and structure shaped by intensive management, artificial selection, and breeding practices [49,50], contrasting significantly with the isolation-driven genetic drift and restricted gene flow currently experienced by the wild northern huemul population. It is possible that observed patterns of moderate levels of inbreeding are the result of a social system in which juveniles are expelled from their family groups and often disperse over extended distances [5,51]. This dispersal behavior would help mitigate the effects of inbreeding and would help maintain genetic diversity in spite of relatively low population size. Nevertheless, these population genetic patterns should be considered with caution and concern. The small number of groups and overall small population size per se could eventually decrease population viability, as has been modeled based on a huemul population in an isolated area with a low number of founder animals [52]. Furthermore, it is also possible that the genetic diversity of the huemul in the Nevados de Chillán will decrease in future generations if no conservation measures are taken to maintain its population size.

### 4.2. Demographic History and Refugial Origin

At a broader timescale, our results could indicate that this huemul population was less impacted by the cold Pleistocene glacial stages than southern populations, and that its small population size could be the result of more-recent events [6]. Similar patterns were detected for the southern river otter (*Lontra provocax*), which also showed evidence of population bottlenecks and low diversity throughout its northern locations [53]. Marín et al. 2013 [6] reported evidence of a recent demographic expansion in this northern huemul population based on mitochondrial DNA and suggested that demographic changes took place during the Pleistocene rather than the Holocene, consistent with the population’s refugial history [6]. Future studies should aim to incorporate high-throughput sequencing approaches (e.g., ddRAD) to provide more robust estimates of genetic variation and inbreeding of this endangered population. However, this will require the collection of higher-quality non-invasive samples, such as hair traps or shed antlers, across a wider area encompassing the Laguna del Laja National Park (Figure 1). Unlike the fecal samples used in the present study, these sample types yield DNA of greater integrity and quantity, which would unlock the full potential of genomic tools for a more comprehensive characterization of this population.

### 4.3. Conservation Threats and Population Viability

Current changes in the northern huemul population are likely the outcome of human activities through habitat destruction, extirpation of populations through hunting, and increased densities of humans [19,46]. Indeed, there have been near-constant losses and fragmentation of the original habitat in the area due to changes in land use, e.g., in areas such as the Valle Las Trancas sector and the sky center, overgrazing by cattle and concomitant disease transmission, dog attacks and the introduction of the exotic deer (e.g., European red deer *Cervus elaphus*, Axis deer *Axis axis*, Dama deer *Dama dama*) have contributed to population declines [13,19] and reduced dispersal [54,55]. Additionally, the construction of large-scale projects such as an oil pipeline (in 1993–1994) and a gas pipeline (in 1992) that crossed part of the primary habitat of the Ñuble National Reserve, where part of maternal diversity is represented today, have likely contributed to the huemul’s decline in the area.

Previous population estimates determined that, on average, the Nevados de Chillán population consists of around 50 individuals confined to an area that represents 27% of their original range and of the available suitable habitat [19]. Furthermore, the nearest huemul population is in Nahuelhuapi National Park (Argentina’s Northern Patagonia), 400 km southeast of Nevados de Chillán [10,51]. Together, the northern huemul’s small population size and its isolation put it at risk of becoming extinct. A conservative demographic simulation suggested a high probability that the Nevados de Chillán population will become extinct within the next 27–42 years [43]. Our demographic simulations show that a census population size of 50 (or smaller) cannot maintain genetic diversity across time, and that a reduction in terms of alleles and genetic diversity is inevitable and can be accelerated if the population size becomes even smaller. Such trends have also been shown to affect other huemul populations [15], other ungulates [54,55], and mammals broadly [56,57].

### 4.4. Effective Population Size and Long-Term Genetic Erosion

The census population size of the Nevados de Chillán huemul population is small. However, the effective population estimated from the genetic variation currently present in the population is relatively high (47, 95% CI 19.2-infinity). Based on an average ungulate effective population size to census population size ratio of 0.2 [2], we can suggest that huemul in the region may have had a population size five times larger than seen today (e.g., 250 individuals). Our population estimate of 47 is in the range of that estimated for red deer (*Cervus elaphus*) populations from Eastern France, Spain, and Norway [58], or white-tailed deer [59]. However, of specific concern is the estimate of an effective population size (Ne) smaller than the modern thresholds recommended to prevent short-term inbreeding depression (Ne ≥ 100) and to maintain long-term evolutionary potential (Ne ≥ 1000) [1,60]. A broader assessment of genetic variation and population size of the regional populations is urgently needed to better model the population dynamics, which ideally includes the southernmost individuals detected from this population in the Laja Lagoon area (37°24′ S).

The most important recent threats to huemul in the area are outside reserves and national parks. For instance, unique genetic forms (e.g., Haplotype 6) that occur in the private “Las Truchas basin” are at risk of disappearing if specific measures are not taken before the implementation of the Punilla Reservoir project, the development of mining in the area, and the construction of an international highway currently being planned. In Central Chile, only three protected areas (the Laguna del Laja National Park, the Ñuble National Reserve, and the Los Huemules del Niblinto National Sanctuary and Reserve) (Figure 1) support the northern huemul population. However, these reserves only represent a portion of the habitat required to conserve and maintain the huemul population of Central Chile.

### 4.5. Conservation and Genetic Management Recommendations

The precarious conservation status of the unique huemul population inhabiting Nevados de Chillán increases the importance of designing and implementing effective and comprehensive management activities that will better monitor, protect, and enhance the long-term conservation efforts. This will require continued protection of populations in public and in private lands, ideally with local community involvement, and in coordination with the support of local, national, and international entities. A more ambitious initiative would include the establishment of a binational program between Argentina and Chile to more broadly protect all huemul populations, as was initiated in 2008. This could be achieved through different management strategies at different levels, including: (1) continuous monitoring of the huemul population and its threats, within the framework of the Nevados de Chillán-Laguna del Laja Corridor, (2) implementation of conservation actions on buffer zones around public and private protected areas which would promote connectivity among huemul groups, and (3) the incorporation of new public policies, and regulations or guidelines that provide greater protection tools for government services.

In terms of genetic management, we specifically recommend (i) establishing a long-term genetic monitoring program using the seven microsatellite loci described here as a quantitative baseline, with repeating surveys every five to ten years to monitor changes in allelic richness, observed and expected heterozygosity, and inbreeding over time, (ii) prioritizing habitat connectivity restoration within the Nevados de Chillán–Laguna del Laja corridor to facilitate natural gene flow among fragmented subgroups, particularly by reducing barriers to dispersal such as cattle fencing and unmanaged grazing areas, and (iii) securing legal protection for key private landholdings outside the existing reserve network, such as the Las Truchas basin where the unique Haplotype 6 occurs, before planned infrastructure projects proceed.

Although future translocation of animals from larger populations in the south may be necessary, these translocations of animals would likely be most effective only after establishing increased habitat protection and restoration, and if done in conjunction with a detailed risk assessment of all program’s stages and analysis of cost/benefits return, as suggested by IUCN [11]. Within the context of a species-wide conservation strategy, it will be important to identify populations with high levels of genetic variation that can be used as sources for translocations of individuals between groups, e.g., from Central Chile towards southern populations that harbor lower genetic diversity or present signs of inbreeding, or which present genetic load. Ultimately, more ecological and genetic studies are urgently needed to better understand huemul to ensure its long-term viability.

## 5. Conclusions

This study provides the first nuclear genetic baseline for the northernmost huemul population in Nevados de Chillán, the most isolated and northerly remnant population of this endangered Andean cervid. Despite extreme geographic isolation, this population retains moderate heterozygosity (H_O_ = 0.296) comparable to larger Patagonian populations, but exhibits high inbreeding (F_IS_ = 0.566), substructure into two clusters with restricted connectivity, and a low effective population size (Ne ≈ 47)—well below the thresholds required to prevent inbreeding depression and preserve long-term evolutionary potential. Demographic simulations indicate that a census size of ~50 individuals cannot sustain current genetic diversity, with allelic richness declining faster than heterozygosity over the next century. Without urgent intervention to restore habitat connectivity within the Nevados de Chillán–Laguna del Laja corridor, secure private lands harboring unique haplotypes, and coordinate binational conservation efforts with Argentina, the long-term viability of this Chilean population remains seriously compromised.

## Figures and Tables

**Table 1:** Summary of the *Hippocamelus bisulcus* samples used in the genetic analyses, including sectors, geographic positions, and total number of samples used from each sector for each genetic marker.

Localities	Geographic Positions	Samples [*n* = 36]
La Quiriquina	36°42′48.53″ S, 71°21′44.92″ O	2
Laguna del Gallo	36°43′44.00″ S, 71°26′14.00″ O	1
La Leonera	36°44′15.19″ S, 71°23′48.49″ O	2
Las Islas	36°44′44.74″ S, 71°25′7.56″ O	1
Cajón Los Baños 1	36°44′45.00″ S, 71°26′04.00″ O	2
Río Santa Gertrudis	36°45′05.00″ S, 71°24′48.00″ O	1
Cajón Los Baños 2	36°45′5.88″ S, 71°26′31.79″ O	1
Mallín del Paco	36°45′55.08″ S, 71°27′14.70″ O	2
El Refugio 1	36°45′56.74″ S, 71°25′14,27″ O	1
El Refugio 2	36°46′02.00″ S, 71°25′15.00″ O	1
La Parva	36°47′0.93″ S, 71° 26′0.82″ O	4
Cajón Nuevo, Río Gato	36°47′8.93″ S, 71°20′49.57″ O	8
Piedra del Carnero	36°47′21.58″ S, 71°26′11.38″ O	3
Relbún	36°58′44.13″ S, 71°27′49.56″ O	2
Las Parías	37°00′2.30″ S, 71°27′51.20″ O	1
Valle Hermoso	37°1′8.25″ S, 71°28′39.72″ O	1
Caitanos	37°1′20.59″ S, 71°26′15.75″ O	3

## Data Availability

The original contributions presented in this study are included in the article. Further inquiries can be directed to the corresponding authors.
